# Hierarchical shared transfer learning for biomedical named entity recognition

**DOI:** 10.1186/s12859-021-04551-4

**Published:** 2022-01-04

**Authors:** Zhaoying Chai, Han Jin, Shenghui Shi, Siyan Zhan, Lin Zhuo, Yu Yang

**Affiliations:** 1 College of Information Science and Technology, Beijing University of Chemical Technology, Beijing, China; 2 School of Public Health, Peking University, Beijing, China; 3 Research Center of Clinical Epidemiology, Peking University Third Hospital, Beijing, China; 4 National Institute of Health Data Science, Peking University, Beijing, China

**Keywords:** BioNLP, Biomedical named entity recognition, Transfer learning, Permutation language model, Conditional random field

## Abstract

**Background:**

Biomedical named entity recognition (BioNER) is a basic and important medical information extraction task to extract medical entities with special meaning from medical texts. In recent years, deep learning has become the main research direction of BioNER due to its excellent data-driven context coding ability. However, in BioNER task, deep learning has the problem of poor generalization and instability.

**Results:**

we propose the hierarchical shared transfer learning, which combines multi-task learning and fine-tuning, and realizes the multi-level information fusion between the underlying entity features and the upper data features. We select 14 datasets containing 4 types of entities for training and evaluate the model. The experimental results showed that the F1-scores of the five gold standard datasets BC5CDR-chemical, BC5CDR-disease, BC2GM, BC4CHEMD, NCBI-disease and LINNAEUS were increased by 0.57, 0.90, 0.42, 0.77, 0.98 and − 2.16 compared to the single-task XLNet-CRF model. BC5CDR-chemical, BC5CDR-disease and BC4CHEMD achieved state-of-the-art results.The reasons why LINNAEUS’s multi-task results are lower than single-task results are discussed at the dataset level.

**Conclusion:**

Compared with using multi-task learning and fine-tuning alone, the model has more accurate recognition ability of medical entities, and has higher generalization and stability.

## Background

Biomedical information extraction is an important tool to handle the unmarked medical literature of exponential growth, and the extracted information has important value for medical research [1]. Biomedical named entity recognition (BioNER) is a basic task in biomedical information extraction to extract interested entities such as diseases, drugs, genes/proteins from complex, unstructured medical texts [2].

With the efforts of many researchers, more and more deep learning networks have emerged, ranging from Convolutional Neural Network (CNN) [3], Long Short-Term Memory Networks (LSTM) [4], to Transformers-based BERT language models in BioNER. But single-task learning has always had the problem of poor generalization in BioNER task. Mehmood and others [5] proposed multi-task learning based on CNN and LSTM to improve the generalization of the model, but the results was difficult to go beyond single-task learning based on Transformers model and unstable. In order to improve the generalization of the model, we do multi-task learning based on Transformers, but the experimental results once again verify that simple multi-task learning results are not stable, some datasets are improved, but some datasets are not better than single-task learning. Therefore, we propose the hierarchical shared transfer learning, which combines multi-task learning with single-task learning, which not only allows the model to have high accuracy, but also improves the generalization and stability of the model.

We used XLNet [6] based on Self-Attention Permutation Language Model (PLM) to replace BERT as encoder in the pre-training phase, avoiding the problem of input noise from autoencoding language model (AutoEncoder LM). When fine-tuning the BioNER task, we decode the output of the XLNet model with conditional random field (CRF) decoder. Because XLNet uses tagged input, the connection layer between XLNet and CRF is tuned with Label [X]. For multi-tasking training, we split the datasets and combined similar entity datasets. We share all the parameters of the XLNet-CRF during training, and then evaluate the effects of each dataset separately. We refer to the model that shares all XLNet-CRF model parameters for multi-tasking learning as MTL-XC. However, the experimental results show that the learning of MTL-XC is unstable. In order to solve this problem, we propose hierarchical shared transfer learning. We divide the parameters of XLNet-CRF model into shared parts and task-specific parts. The shared portion is for multi-task learning, the specific task portion is for single-task learning, and we refer to this new model as MTL-LS, with good results.

- Permutation language model and conditional random field were combined.
- Aiming at the instability of multi-task learning in BioNER, a hierarchical shared transfer learning method combining multi-task learning and single-task learning was proposed.
- Through the analysis of the physical relationship between the training set, the test set and the training effect, the source of the data-level error was obtained.

The source code is detailed in : https://github.com/pwldj/MTL-BioNER.

## Related work

### Transfer learning

Transfer learning has gained general attention in the field of machine learning in recent years [7] by transferring knowledge from relevant tasks that have been learned to improve new tasks [8]. Transfer learning can be divided into instance-based transfer, feature representation transfer, parameter transfer and relational knowledge transfer [9]. Where parameter transfer is already commonly used in NLP tasks, it is assumed to share some parameters between source tasks and target tasks, or to share a prior distribution of model hyperparameters [10]. This also enables good accuracy when transferring the original model to the new domain [11, 12]. However, there are also problems with negative transfer. For the problem of negative transfer, Wang et al. quantifies the similarity between target domain and source domain by calculating the affinity matrix of gene, and automatically learns the fusion network of target cancer [13]. Tao et al. proposes that the REFERENCE algorithm makes use of the semantic correlation between source sample and target task, rather than the task/sample similarity [14].

### Multi-tasking

Transfer learning, which can be attributed to making the most of all available information, has become an important research direction in Biomedical named entity recognition [15, 16]. Multi-tasking learning (MTL) [17] is a major form of transfer learning that involves learning part of a model or the whole on multiple similar tasks, thereby enhancing the recognition of the model on a particular type of task. Crichton et al., first applied it to the field of BioNER, and by using convolutional neural networks and different shared layer methods, it achieved more than single-task learning (STL) on some entity types [18]. However, the performance on the remaining entity types was not satisfactory. Then, the LSTM gradually became the mainstream of BioNER [19]. Wang et al. [20] achieved an improvement over single-task learning by sharing different parameters embedded in words and character levels. Mehmood et al. use stack-LSTM to share underlying LSTM to multiple similar tasks, while upper-level LSTM trains for different tasks [5]. However, Zuo and Zhang train as a shared layer except for CRF, which trains separately for each task [21].

### Fine-tune

Fine-tuning is another way of transferring learning, pre-training a large amount of unseen data before applying the pre-training model to other specific downstream tasks. Fine-tuning greatly promotes the study of natural language processing [22, 23], and the multi-head self-attention mechanism solves the disadvantages of the unidirectional LSTM model [24]. The BERT language model based on multi-head attention mechanism proposed by Devlin has achieved the most advanced results in many tasks [25]. The BioBERT model and the PubMedBert model have achieved significant improvement in many biomedical tasks by pre-training BERT models using medical materials and fine-tuning them [26, 27]. Based on the BERT model, multi-task learning is used to train multiple medical text mining tasks. But it has also been found that multi-task is not always effective [28]. But the BERT model produces noise during the pre-training phase that reduces the recognition of each word element. The XLNet language model improves the pre-training process of the BERT model [6]. XLNet combines the autoencoder and autoregressive language models and proposes that the PLM can effectively suppress mask noise by predicting the different permutation of the same input sequence. XLNet outperformed the BERT model on 20 natural language processing tasks. The effectiveness of the Distributional Hypothesis of XLNet can acquire common sense and the structure of language from the statistical law of corpus. Its modeling approach from “unidirectional” context to “bidirectional” context and from “short-range” dependency to “long-range” dependency makes XLNet the most refined model for context modeling today. So our research revolves around XLNet.

## Materials and methods

### XLNet-CRF architecture

XLNet is a permutation language model. In the pre-training phase, the noise in BERT model is eliminated by using the two-stream self-attention. At the fine-tuning phase, there is little difference between XLNet and the BERT model, both of which can be considered multi-headed self-attention language models.

**Fig. 1:**
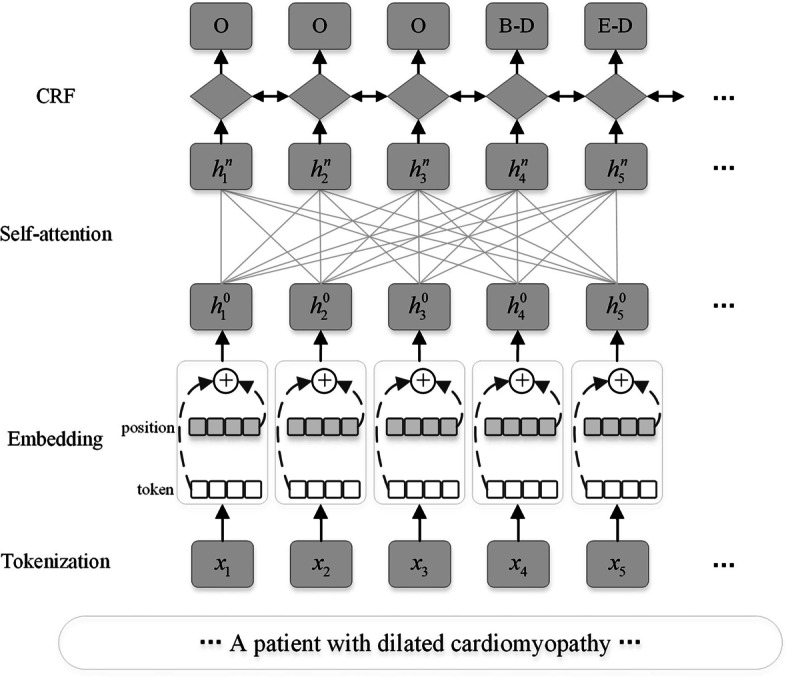
Architecture of the XLNet-CRF model

We have decoded the output of the XLNet model using the CRF decoder. Figure 1 shows the XLNet-CRF architecture from a fine-tuned perspective. First, the text is serialized, and the input sequence is defined as $X=[x_1\dots x_t]$, where *t* is the length of the input sequence. The input sequence in XLNet is generated by the SentiencePiece [20] based on the input text. Then, after the *X* has been word-embedded, each input character is mapped to a vector, forming the sequence $H^0=[h_1^0\dots h_t^0]$ as input to the multi-header attention model. Finally, the output vector of the final XLNet model is $H^n=[h_1^n\dots h_t^n]$ after the attention model is linked by the *n* layer residue. The entity label for each character entered, corresponding to the input, is treated as $Y=[y_0\dots y_t]$. Defines an entity label collection as $l\in {1,2\dots L}$, *L* is the total number of target identification tag sets, so BioNER tasks can be considered classification tasks that predict *Y* based on *X*. Given the continuity of entity labels, the CRF is used as the decoding layer to select the most appropriate label from the label collection. *A* is defined as a transition matrix to modify the current forecast based on previous label information. Therefore, the label forecast score is defined a

1 $$\begin{aligned} \begin{aligned} S_{(X,y)}=\sum _{i=0}^LA_{y_i,y_i+1}+\sum _{i=1}^LP_{i,y_i} . \end{aligned} \end{aligned}$$

After softmax standardizes the label score, the conditional probability for each word element can be obtained. At the prediction and evaluation stage, the Viterbi algorithm [29] is used as the reasoning for the final prediction results.

### Multi-task learning

The general deep learning model can fit the training target data highly. However, even for datasets of the same type of entity, it is still difficult to apply models trained for one dataset directly to another dataset [30]. On the one hand, when only one data set is targeted, there is inevitably an out-of-vocabulary (OOV). To some extent, the problem of OOV is mitigated by the paraphrasing of sentences. On the other hand, over-fitting is common for models that train on only one dataset. With the limited size of a single dataset, multi-tasking learning is an effective way to improve the generalization of models, while avoiding the over-fitting of models trained on a single dataset. Multi-tasking is the training of shared parameters on multiple tagged datasets where similar entities exist. In this paper, fine-tuning is integrated into multi-task learning. Pre-trained XLNet with common text data is used as an initialization parameter, while CRF model parameters are initialized randomly. On this basis, similar entity datasets are combined for training and the effects of each dataset are evaluated separately, corresponding to which single task learning is defined as fine-tuning training for each dataset on a pre-training model basis. We divided 14 datasets into four categories for multi-task learning. We share all the parameters of XLNet-CRF for multi-task, and we call this model MTL-XC.

### Hierarchical shared transfer learning

We trained in single-task learning and multi-task learning respectively to evaluate the effectiveness of multi-task learning and found that the results were not as good as expected.

**Fig. 2:**
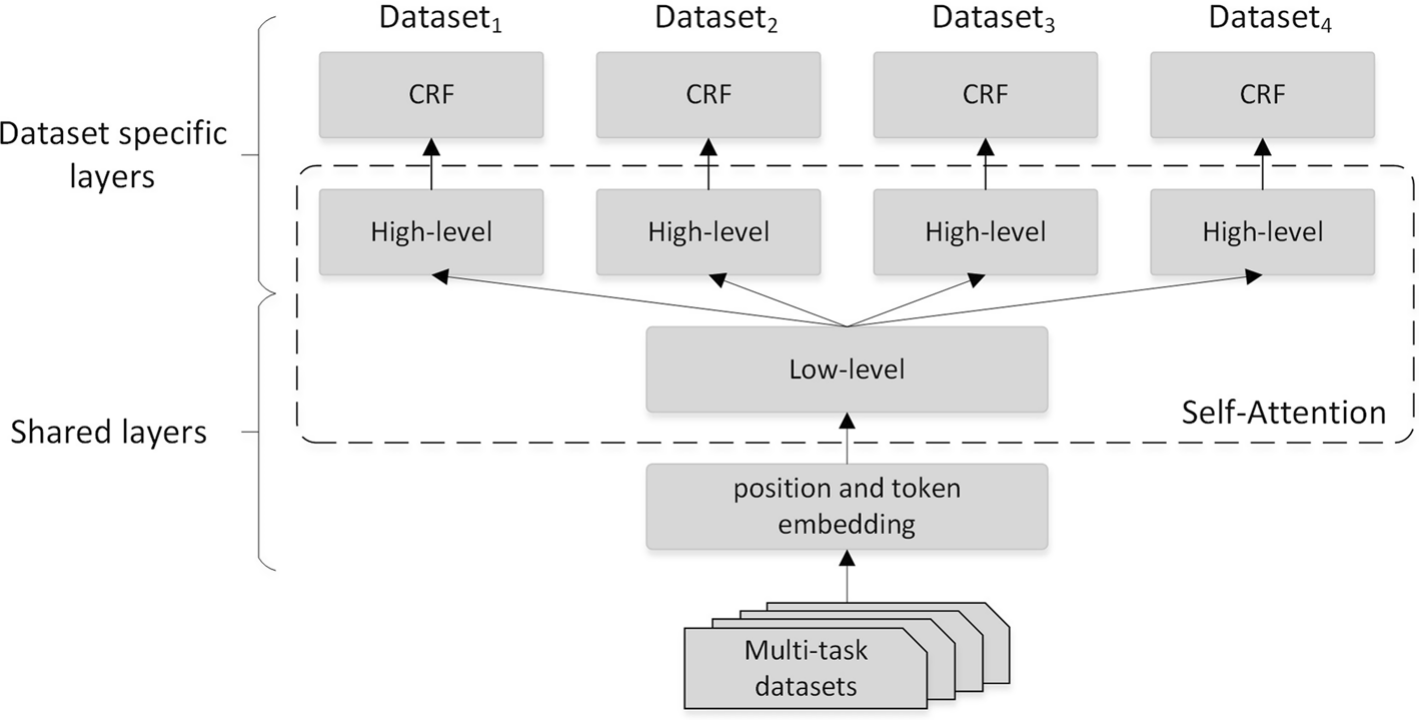
Model segmentation schematics for hierarchical shared transfer learning. We split XLNet-CRF. The underlying layers of the token embedding and XLNet models are share, with the upper layers and CRFs of the XLNet as specific tasks

Inspired by the work of Mehmood et al., we proposed the MTL-LS (layer slicing) model. As shown in Fig. 2, we divide the XLNet-CRF model into shared and task-specific sections by layer. Fortunately, the parameters in the hidden layers of the XLNet model have the same output size, allowing them to be split and combined at will. Take $H^k$ as the dividing point between $H^0$ and $H^n$, define the underlying layer between 0 *k*, and the layer between *k* *n* is called the upper layer. Because the underlying contains the underlying text encoding information [31], we use the underlying as a shared layer, and the upper layer is a special task layer trained separately for different tasks. The underlying parameters are derived from the corresponding layer parameters of the MTL-XC training. The upper layer parameters are initialized by pre-trained parameters on the common corpus, which can accelerate the convergence of the model. CRF contains few parameters ($L^2$) and is closest to the decoding layer, making it easier to train, so random initialization is still used so that the decoder can train the language characteristics of different tasks separately. It encodes and decodes specific tasks and retains the common encoding information learned by multi-task learning for a class of entities. Define the scale of the number of shared layers after split as the slicing rate ($slicing rate=k/n$), and when $slicing rate=0$, MTL-LS degrades to the single-task learning that is shared by the embedded table parameters. When $slicing rate=0$, this is similar to the method proposed Zuo and Zhang [21], where the model is divided into two parts: the encoder (XLNet) and the decoder (CRF). The encoder part is used as a shared layer, and the decoder part is used for specific datasets.

### Datasets and data preprocessing

Using datasets similar to those in Crichton et al. [18]. We excluded AnatEM during the hierarchical shared transfer learning phase because the dataset was not in the 4 types of entities ultimately evaluated. Furthermore, we experimented on 14 other baseline datasets and divided the entities into four categories: gene/protein, chemical, disease, and species. We take BC5CDR [32], BC4CHEM [33], NCBI-disease [34], BC2GM [35]and LINNAEUS [36], five datasets are gold standard master datasets. We analyzed the relationship between training sets, test sets, and training effects for five gold standard datasets. These datasets are open and available from https://github.com/cambridgeltl/MTL-Bioinformatics-2016.

### Evaluation metrics

Due to the limitation of the training cost, it is difficult to conduct multiple random initialization training, so instead, for each dataset, we first conducted *n* epoch training sessions and then conducted $k*m$-round epoch training sessions with the obtained model parameters as the starting point. With the increment of *n*, *m*, the time cost becomes unacceptable and the convergence effect of the model has not been significantly improved. However, if *n*, *m* is too small, also cannot converge. And finally we took a $30+3*30$ structure to train. In the test, the last five checkpoints of each training exercise were predicted against the test datasets. We calculated precision, recall, and F1-scores as evaluation indicators, with F1-scores as the primary evaluation indicators. The calculation formula is as follows:

2 $$\begin{aligned} F1-score= & {} \frac{2\cdot precision\cdot recall}{precision+recall} \end{aligned}$$

3 $$\begin{aligned} precision= & {} \frac{TP}{TP+FP} \end{aligned}$$

4 $$\begin{aligned} recall= & {} \frac{TP}{TP+FN} \end{aligned}$$

### Training detail

XLNet-Large pre-training parameters provided by Yang et al. [6], which contain 24 layers, 1024 hidden layer nodes, and 16 attention heads. We’re using Adam optimizer, Adam epsilon was $1e-6$ and the learning rate was $3e-5$, because the model was already pre-trained, so the model was not warmed-up during training. When multi-tasking, we unify the labels of the datasets and shuffle the datasets. In order to preserve the underlying shared information as much as possible, the layer attenuation strategy is used to reduce the learning rate of each layer, i.e. $lr[n-1]=lr[n]*decay\_rate$, where *n* is the XLNet layer, and $decay\_rate=0.9$.

## Results

### Share all the parameters of the XLNet-CRF (MTL-XC)

In this lab, the effects of four types of BioNER in MTL-XC were evaluated. Benchmark the results of training on a single-task. For datasets that have multiple entity types, we compare them separately into a single type. Table 1 provides a complete comparison of the performance of chemical, disease, species, and gene/protein on MTL-XC’s precision, recall, F1-scores for 14 datasets. As can be seen from Table 1, the F1 of the two types of entities, disease, and gene/protein, has been greatly enhanced, and in the vast majority of the datasets, multi-task is better than single-task. Some datasets have been improved significantly, such as BioNLP13GE dataset by 5.37, Ex-PTM dataset by 6.73 and CRAFT dataset by 3.69. Although there has been an increase in disease entities, the increase has been relatively limited. In the remaining datasets, performance declines are severe. The same phenomenon occurs in the species category, where all datasets have a lower MTL-XC results than single-task learning. Therefore, direct sharing of full model parameters is not ideal. Again, this proves that multi-tasking is not always better than single-task learning [28]. Entity categories and dataset features affect multi-tasking learning results, including association between dataset and size of datasets.

**Table 1:** Performance of STL-DS and MTL-XC on all tasks

Dataset	STL			MTL-XC		
	P. %	R. %	F1	P. %	R. %	F1
*Chemical*						
BC4CHEMD	93.00	92.40	**91**.**70**	92.02	92.49	91.25
BC5CDR	92.76	93.96	**93**.**36**	93.43	92.94	93.19
BioNLP11ID	55.56	72.58	62.94	61.44	75.81	**67**.**87**
BioNLP13CG	83.20	85.14	**84**.**16**	82.35	80.88	81.61
BioNLP13PC	88.57	90.33	**89**.**44**	76.65	83.80	80.07
CRAFT	84.07	81.05	**82**.**54**	75.34	72.38	73.83
*Disease*						
BC5CDR	84.83	88.11	86.44	86.40	87.34	**86**.**87**
NCBI-disease	87.27	89.27	88.26	87.80	89.73	**88**.**75**
*Gene and protein*						
BC2GM	81.91	82.53	82.22	82.67	82.61	**82**.**64**
BioNLP09	88.20	86.82	87.50	87.23	91.95	**89**.**53**
BioNLP11EPI	84.23	87.96	85.81	85.32	87.63	**86**.**46**
BioNLP11ID	89.22	89.65	**89**.**43**	89.6	88.47	89.03
BioNLP13CG	88.45	92.42	90.39	93.56	91.63	**92**.**58**
BioNLP13GE	73.57	83.51	78.22	77.62	90.55	**83**.**59**
BioNLP13PC	89.56	94.26	**91**.**85**	90.66	87.93	89.27
CRAFT	80.48	75.44	77.88	78.56	84.83	**81**.**57**
Ex-PTM	74.79	80.46	77.52	81.83	86.83	**84**.**25**
JNLPBA	71.98	80.04	75.80	72.58	85.04	**78**.**32**
*Species*						
BioNLP11ID	85.41	82.03	**83**.**68**	91.22	70.24	79.37
BioNLP13CG	88.34	89.19	**88**.**76**	88.39	86.68	87.52
CRAFT	96.45	97.73	**97**.**08**	93.76	93.51	93.63
LINNAEUS	91.70	85.62	**88**.**56**	88.43	82.14	85.17

Better scores of each metric are in bold

### Hierarchical shared transfer learning on XLNet-CRF (MTL-LS)

We try to further improve the training effectiveness and stability of multi-task learning by layering the model. The new model is referred to as MTL-LS, as detailed in “Hierarchical shared transfer learning” section. Based on previous MTL-XC studies, we further trained fourteen datasets on MTL-LS. We made *slicingrate* 0.25, 0.50, 0.75, and 1.00, respectively, as shown in Table 2.

**Table 2:** F1 performance of STL, MTL-XC, and MTL-LS with different slicing rates

Dataset	STL	MTL-XC	Slicng rate			
			0.25	0.50	0.75	1.00
*Chemical*						
BC4CHEMD	91.70	91.25	91.82	92.24	92.37	**92**.**47**
BC5CDR	93.36	93.19	**93**.**93**	93.75	93.60	93.14
BioNLP11ID	62.94	67.87	42.29	52.98	73.41	**73**.**71**
BioNLP13CG	84.16	81.61	78.59	78.50	**85**.**13**	82.47
BioNLP13PC	**89**.**44**	80.07	81.25	82.13	84.03	86.00
CRAFT	**82**.**54**	73.83	76.73	79.74	77.96	79.44
*Disease*						
BC5CDR	86.44	86.87	86.10	87.04	86.28	**87**.**34**
NCBI-disease	88.26	88.75	85.23	86.79	**89**.**24**	88.97
*Gene and protein*						
BC2GM	82.22	82.64	79.85	81.54	81.94	**82**.**94**
BioNLP09	87.50	89.53	87.23	**89**.**53**	89.47	89.27
BioNLP11EPI	85.81	86.46	84.65	85.65	86.24	**86**.**58**
BioNLP11ID	89.43	89.03	83.62	82.69	**89**.**59**	86.55
BioNLP13CG	90.39	92.58	91.33	92.05	**92**.**71**	92.21
BioNLP13GE	78.22	83.59	81.29	82.61	82.73	**84**.**03**
BioNLP13PC	91.85	89.27	89.16	**91**.**87**	90.59	90.37
CRAFT	77.88	81.57	79.02	82.62	**83**.**42**	82.18
Ex-PTM	77.52	84.25	78.52	80.57	**84**.**64**	84.55
JNLPBA	75.80	**78**.**32**	75.08	77.00	77.32	77.66
*Species*						
BioNLP11ID	83.68	79.37	70.91	77.45	**83**.**74**	79.00
BioNLP13CG	88.76	86.30	**88**.**97**	87.39	86.07	86.90
CRAFT	**97**.**08**	93.63	95.56	95.04	94.74	95.75
LINNAEUS	**88**.**56**	85.17	83.82	84.05	86.40	85.06

Better scores of each metric are in bold

We can see that the effects of different datasets are not stable with *slicingrate*. BC5CDR-chemical dataset results best when $Slicing rate=0.25$, but $Slicing rate=1.00$ in BC4CHEMD, which is also a chemical entity. BioNLP13PC-chemical and CRAFT-chemical did not exceed single-task learning results but had a 5.93 and 5.91 improvement over multi-tasking, respectively. For disease entities, the BC5CDR-disease dataset has the largest F1 value at $slicing rate=1.00$, and the NCBI-disease dataset has the best effect at $slicing rate=0.75$. MTL-LS is better than single-tasks in all 10 sub-tasks of the gene/protein entity. In the species class, BioNLP11CG and CRAFT obtained the best results at the *slicingrate* of 0.25 and 1.00 respectively, while BioNLP13ID and LINNAEUS obtained the best results at the *slicingrate* of 0.75. However, CRAFT-species and LINNAEUS dataset are slightly different, although the F1 value is higher than the MTL-XC, but they do not reach the F1 value achieved by single-task learning. In “${card (C_L\cap P\cap T)/card (C_L\cap P)} {\text{ and }} (cardP-cardL)/cardL$” sections, we analyze the five gold standard dataset to find that the LINNAEUS data is of lower quality and smaller size, and that the introduction of other entity sets for training would reduce its F1 value.

The results show that MTL-LS is greatly affected by *slicingrate* and in some cases even learns less than STL. On the one hand, as *slicingrate* decreases, models converge more and more slowly. On the other hand, the relevance of the data itself makes it possible for the model to learn redundant noise information. But finding the right *slicingrate* makes the result except for JNLPBA better than all results on MTL-XC. Overall, training using MTL-LS resulted in a certain degree of steady improvement relative to MTL-XC. Sharing mechanisms at different levels can make it easier for a model to jump out of a local best.

Among the five sub-tasks for four gold standard datasets excluding LINNAEUS, the model presented in this paper has a significant advantage over the model based on LSTM [18, 37]. BC5CDR-chemical, BC5CDR-disease, BC2GM, BC4CHEMD, NCBI-disease compared to single-task XLNet models increased by 2.81, 1.67, 1.52, 1.18, 0.97 and 1.3 percentage points respectively. It can be concluded that the proposed MTL-LS architecture has better effect, generalization, and stability on BioNER.

### Comparison with benchmark results

In this section, compare the results of MTL-LS for the five gold standard datasets with those of other the corresponding publications. The datasets we use are standard that already publicly available, so test splits are the same. To make a fair comparison with other people’s work, we adjust the *slicingrate* on the development set of the data set and produce the final test set results, so that the *slicingrate* is not optimized on the test set. Take test set F1-score as shown in Table 3.

**Table 3:** Model performance comparison to other studies

	BC5CDR		BC2GM	BC4CHEMD	NCBI-disease	LINNAEUS
	Chemical	Disease				
Crichton et al. [18]	89.22	80.46	73.04	82.95	80.46	83.98
Yoon et al. [37]	93.31	84.08	79.73	88.85	86.36	–
Lee et al. [26]	93.44	86.56	**84**.**40**	91.41	**89**.**36**	**89**.**81**
BERT [26]	91.16	82.41	81.79	90.04	85.63	87.60
PubWebBERT [27]	93.33	85.62	84.52	–	87.82	–
HunFlair [38]	–	–	–	–	88.65	–
STL	93.36	86.44	82.52	91.70	88.26	88.56
Proposed	**93**.**83**	**87**.**28**	82.92	**92**.**42**	89.25	86.37

Better scores of each metric are in bold

In general, the methods presented in this paper perform poorly on the LINNAEUS dataset. After analysis, the model can easily converge with the local optimal when training LINNAEUS and has not found a way to improve the effect. Except LINNAEUS, single-task training results were better than the BERT. The XLNet-CRF single-task is not good enough compared to PubWebBERT with only BC2GM. NCBI-disease is better at STL without HunFlair, but MTL-LS is better than HunFlair. BioBERT achieved more than single-task training through further pre-training in medical data and exceeded the model proposed in this paper on BC2GM, NCBI-release and LINNAEUS. However, hierarchical shared transfer learning outperforms BioBERT models on BC5CDR and BC4CHEMD datasets. It can be argued that pre-training of knowledge data in biomedical fields can significantly improve entity identification of genes/proteins and species classifications. For other entity types, it is better to combine multitasking with fine-tuning.

## Discussion

**Fig. 3:**
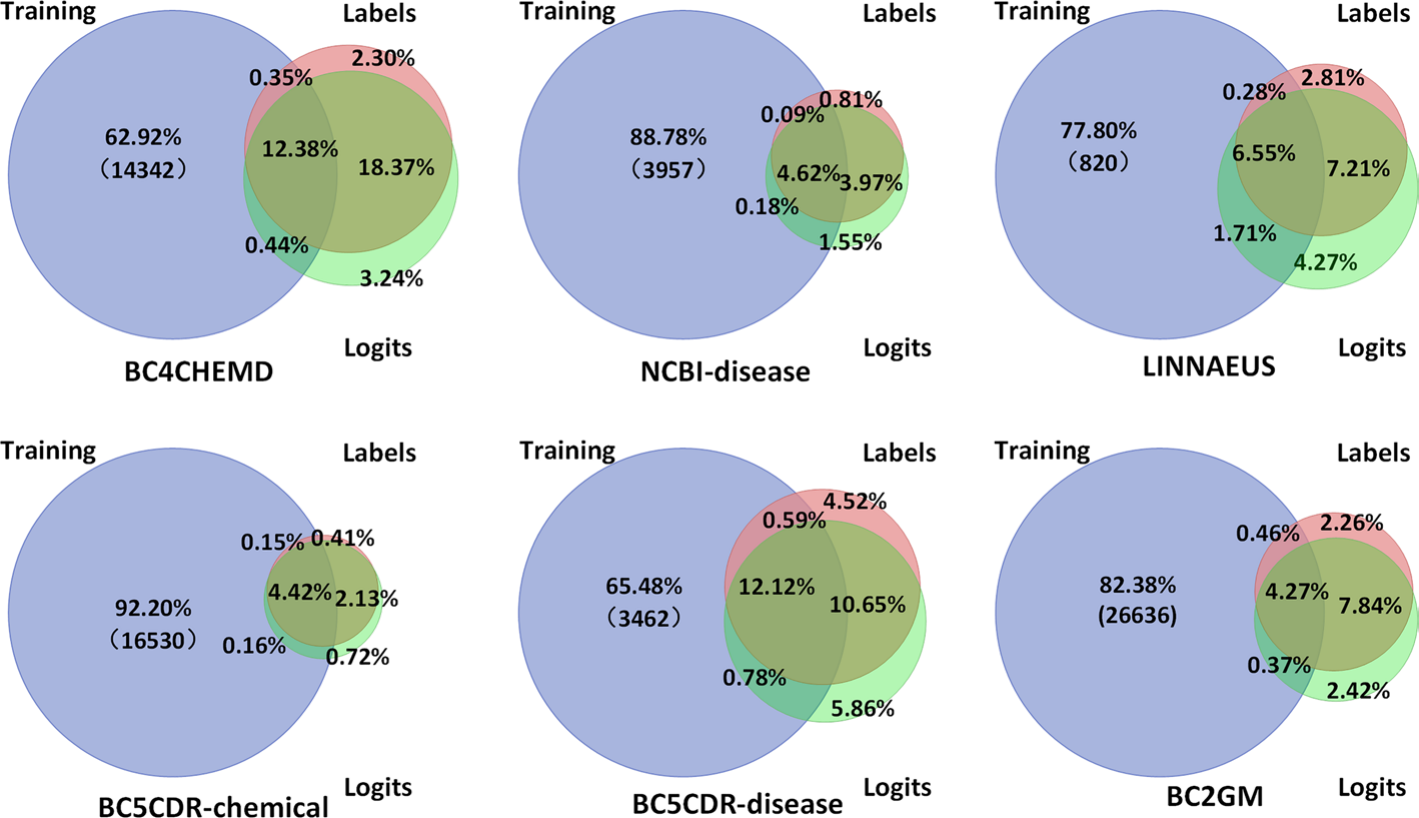
Euler diagram of training entity set (Training), test set (Labels) and predict set (Logits)

**Fig. 4:**
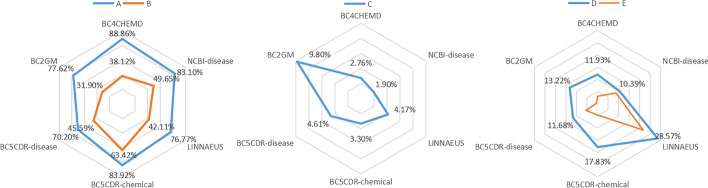
Radar chart of the proportional relationship between the sets of six tasks. **A** $card(C_T\cap L\cap P)/card(C_T\cap L)$, **B** $card(T\cap L)/cardL$, **C** $card(C_P\cap T\cap L)/card(T\cap L)$, **D** $card(C_L\cap P\cap T)/card(C_L\cap P)$, **E** $(cardP-cardL)/cardL$

Multi-task learning essentially increases the generalization of the model by increasing the number of training samples to cover as many entities as possible. Therefore, the correlation of data in multi-task learning often greatly affects the effect of training. If the data is less dependent, the F1 value is lowered. We counted the entities of five dataset, where the multi-task learning entity set Training is represented by *T*, and the test set contains the entity set Labels represented by *L* and the entity set Logits predicted by the final model are represented by *P*, shown in Fig. 3 as a Euler graph. We compute the number of parts of the set, shown in Fig. 4 as a radar chart.

### $card(C_T\cap L\cap P)/card(C_T\cap L)$

We take BC4CHEMD as an example to describe the meaning of the Euler diagram, and $C_T\cap L$ (2.30%+18.37%) indicates a set of entities that need to be predicted but not trained. $C_T\cap L\cap P$(18.37%) denotes the set of entities that are not in the training set, but that predict success. The greater the $card(C_T\cap L\cap P)/card(C_T\cap L)$, the more adaptable the model is. We present the $card(C_T\cap L\cap P)/card(C_T\cap L)$ of the six datasets as a radar chart in Fig. 4A. The figure shows that this percentage of BC4CHEMD reached 88.86%, which explains why the F1 value of BC4CHEMD can reach 92.47%. The average value of $card(C_T\cap L\cap P)/card(C_T\cap L)$ can reach 80.08%, which is a good indication that our model learns a certain amount of knowledge, has some learning ability, and can transfer learning.

### $card(T\cap L)/cardL$

Figure 4B indicates that $card(T\cap L)/cardL$, $T\cap L$ represents the intersection of the training set and the test set entities. The greater this value, the greater the proportion of entities covered by the training set, the greater the generalization of the model. The average can reach 45.13%, BC5CDR-chemical $card(T\cap L)/cardL$ even reached 63.42%.

### $card(C_P\cap T\cap L)/card(T\cap L)$

Figure 4C shows $card(C_P\cap T\cap L)/card(T\cap L)$, where $C_P\cap T\cap L$ is the set of entities that have been learned by the training set but cannot be identified in the test set, which represents an average of 4.42% of $T\cap L$. This phenomenon suggests that even learned knowledge can be forgotten, and the introduction of a larger number of samples can distract the model, resulting in a loss of memory and a lack of recognition of the learned entity. We can see from the radar chart that the value of card $card(C_P\cap T\cap L)/card(T\cap L)$ for BC2GM reached 9.8%. Second, BC2GM mixes ten task genes/protein entities of $card(T\cap L)/cardL$, (Fig. 4B) and only 31.90%. For BC2GM, which forgets 9.8% of the knowledge and has low coverage, the F1 value is the worst in the six datasets.

### $card(C_L\cap P\cap T)/card(C_L\cap P)$

Another particular note is the radar chart shown in $card(C_L\cap P\cap T)/card(C_L\cap P)$ in Fig. 4D. $C_L\cap P$ represents the set of entities that recognize the error, and $C_L\cap P\cap T$ and $C_L\cap P\cap C_T$ are included in $C_L\cap P\cap T$, where $C_L\cap P\cap T$ is very noteworthy. The entity set is treated as the entity training in the training set, but in the test set $C_L\cap P\cap T$ is not the entity, that is, the wrong knowledge is learned when learning, or the model identifies the accurate entity according to the prior knowledge, and the test set tells the model that the prediction error is contradictory. Therefore, the larger $C_L\cap P\cap T$ as $C_L\cap P$, the worse the quality of the dataset. Figure 4D shows that this problem exists in all six tasks, with an average of 15.6%. That is, the dataset itself has some errors. And the LINNAEUS dataset has 28.57% error recognition from training data, which is why LINNAEUS’s single-task results are much better than multi-task results. The introduction of new training samples increases the probability of model error. The relevance of datasets in species categories can be considered to have contributed to this result. There is a need to improve the quality of data for this type of dataset to avoid problems with misperception.

### $(cardP-cardL)/cardL$

As shown in Fig. 4E, the $(cardP-cardL)/cardL$ values are greater than 0, that is, the number of entities predicted by all sets is greater than the original number of entities in the test set. The LINNAEUS dataset has a value of 21.64%, which again explains why the LINNAEUS multi-task F1 value never reaches the effect of a single-task. For LINNAEUS, our model has learned a lot that is not its own.

*In conclusion,* we analyze the relationship between training sets, test sets, and entity sets of predicted results, and point out the error sources at the data level. This paper explains the reason the experiment results of LINNAEUS dataset can’t transcend the single-task at the data level.

## Conclusion

Because of the previous methods of biomedical named entity recognition through deep learning methods, single-task learning or multi-task learning. This paper presents an effective hierarchical shared transfer learning method, which combines multi-task with single-task, has high generalization and stability, and validates its effectiveness on fourteen datasets. In addition, we analyzed the physical relationship between training sets, test sets, and training effects on five gold standard datasets. The source of error at the data level is pointed out.

## Data Availability

XLNet-Large pre-training parameters were provided by [https://storage.googleapis.com/xlnet/released_models/cased_L-24_H-1024_A-16.zip]

## Abbreviations

BioNER: Biomedical named entity recognition
CRF: Conditional random field
CNN: Convolutional neural networks
BiLSTM: Bidirectional long short-term memory
BERT: Bidirectional encoder representations from transformers
AutoEncoder LM: AutoEncoding language model
OOV: Out-of-vocabulary
MTL: Multi-task learning
STL: Single-task learning
MTL-XC: Share all the parameters of the XLNet-CRF
MTL-LS: Hierarchical shared transfer learning on XLNet-CRF
